# The Effects of Anthocyanin-Rich Bilberry Extract on Transintestinal Cholesterol Excretion

**DOI:** 10.3390/foods10112852

**Published:** 2021-11-18

**Authors:** Jimin Hong, Minji Kim, Bohkyung Kim

**Affiliations:** Department of Food Science and Nutrition, Pusan National University, Busan 46241, Korea; hongjm17@pusan.ac.kr (J.H.); minjikim17@pusan.ac.kr (M.K.)

**Keywords:** bilberry, transintestinal cholesterol excretion, LDL receptor, hypocholesterolemia, Caco-2 cells

## Abstract

Hypercholesterolemia is one of the modifiable and primary risk factors for cardiovascular diseases (CVD). Emerging evidence suggests the stimulation of transintestinal cholesterol excretion (TICE), the nonbiliary cholesterol excretion, using natural products can be an effective way to reduce CVD. Bilberry (*Vaccinium myrtillus* L.) has been reported to have cardioprotective effects by ameliorating oxidative stress, inflammation, and dyslipidemia. However, the role of bilberry in intestinal cholesterol metabolism is not well understood. To examine the effects of bilberry in intestinal cholesterol metabolism, we measured the genes for cholesterol flux and de novo synthesis in anthocyanin-rich bilberry extract (BE)-treated Caco-2 cells. BE significantly decreased the genes for cholesterol absorption, i.e., Niemann-Pick C1 Like 1 and ATP-binding cassette transporter A1 (ABCA1). In contrast, BE significantly upregulated ABCG8, the apical transporter for cholesterol. There was a significant induction of low-density lipoprotein receptors, with a concomitant increase in cellular uptake of cholesterol in BE-treated cells. The expression of genes for lipogenesis and sirtuins was altered by BE treatment. In the present study, BE altered the genes for cholesterol flux from basolateral to the apical membrane of enterocytes, potentially stimulating TICE. These results support the potential of BE in the prevention of hypercholesterolemia.

## 1. Introduction

Hyperlipidemia, including hypercholesterolemia and hypertriglyceridemia, is a modifiable risk factor for atherosclerosis and cardiovascular disease (CVD) [1]. Disrupted cholesterol balance is highly associated with hyperlipidemia. The concentration of plasma cholesterol is tightly regulated by cellular cholesterol homeostasis [2]. Cholesterol homeostasis is maintained by the interplay of the liver and the intestine [2,3]. The liver, a central site for cholesterol homeostasis, plays a primary role in de novo biosynthesis, assembly and uptake of cholesterol, and conversion of cholesterol to bile acids for biliary secretion. The function of the intestine in cholesterol homeostasis is the absorption of dietary cholesterol and biliary cholesterol [4]. Cholesterol excretion from the body is crucial in cholesterol homeostasis in mammals, as there is no mechanism for cholesterol degradation other than conversion to bile acids [5]. Classically, the reverse cholesterol transport pathway driven by high-density lipoprotein has been recognized as the only route to remove cholesterol from the body. In this hepatobiliary cholesterol excretion pathway, the cholesterol is secreted to bile from the liver for subsequent excretion as a fecal neutral sterol [4,5,6]. However, accumulating evidence suggests that the intestine may play an unanticipated role in transintestinal cholesterol excretion (TICE), the second major pathway for cholesterol excretion [7,8,9]. Enterocytes can directly take up circulating lipoprotein-derived cholesterol from plasma for subsequent disposal into the intestinal lumen in this alternative pathway for cholesterol elimination [10,11]. The underlying mechanistic information of this alternative route for cholesterol elimination remains largely unknown. However, several studies reported that TICE accounts for 30–40% of fecal neutral sterol excretion in both mice and humans under normal conditions [11,12]. Furthermore, this nonbiliary cholesterol excretion pathway can be stimulated by Liver X receptors (LXRs), peroxisome proliferator-activated receptor delta (PPARδ), farnesoid X receptor, ezetimibe, and plant sterols [13,14,15,16,17,18,19]. Therefore, targeting the direct contribution of TICE to fecal neutral sterol secretion can provide an effective strategy for the prevention of CVD.

Polyphenols, particularly anthocyanin-rich foods, have been reported to have cardioprotective properties [20,21,22]. Bilberry (*Vaccinium myrtillus* L.), often known as the European blueberry, is one of the rich sources of polyphenols and anthocyanins. It has been widely used as folklore medicine for diarrhea, dysentery, scurvy, diabetes, stroke, and mouth and throat inflammation [23]. It has been commonly used as a dietary and supplement ingredient to alleviate oxidative stress, inflammation, and ocular health [24]. Bilberry has been reported to have protective effects against oxidative stress, inflammation, hyperglycemia, and lipid metabolism, which are associated with the initiation and progression of CVD [25,26,27,28,29,30,31,32,33]. The effects of bilberry on hypercholesterolemia have been reported in vitro, in vivo in animals, and in clinical trials [33]. Bilberry supplementation improved serum cholesterol profiles in high-fat-fed mice, Fischer rats, aging perimenopausal rats, Zucker diabetic fatty rats, and alloxan-induced diabetic rats [34,35,36,37,38]. Consumption of bilberry improved cholesterol profiles and concentrations in hypercholesterolemic subjects in clinical trials [39,40]. Despite these promising results in animal models and clinical trials, little is reported about the effects of bilberry on intestinal cholesterol metabolism. Studies reported that the effects of bilberry on hypercholesterolemia could be attributed to its high content of polyphenols and anthocyanins [41]. In addition, natural products with high concentrations of polyphenols stimulated the TICE pathway by altering the genes involved in cholesterol flux from the basolateral to the apical membrane of enterocytes [42,43,44,45]. In the present study, we investigated the effects of BE in regulating genes involved in intestinal cholesterol metabolism using Caco-2 cells.

## 2. Materials and Methods

### 2.1. Total Anthocyanin, Phenolic, and Flavonoid Contents

Anthocyanin-rich bilberry extract (BE), the spray-dried and standardized bilberry extract to 25% anthocyanins (cyanidin-3-glucoside), was kindly provided from Artemis International (Fort Wayne, IN, USA). Total anthocyanin content was examined by the pH differential method of Giusti and Wrolstad [46]. Briefly, 75 μL of 1 mg/mL BE was mixed with 1425 μL of 0.025 M potassium chloride buffer (pH 1.0) and 0.4 M sodium acetate buffer (pH 4.5), and incubated for 15 min at 23 °C. The absorbance of the mixture was measured at 510 and 700 nm using a microplate reader. Total anthocyanin contents of BE were expressed as mg cyanidin-3-glucoside equivalents (CGE)/100 g dried weight. Total phenolic and total flavonoid amount was measured using Folin and Ciocalteu’s reagent and a modified method, as previously described [47]. To measure the phenolic contents, 10 μL of BE sample was added to 130 μL of deionized water, and subsequently 10 μL of Folin and Ciocalteu’s reagent was added for a 6 min reaction. Then, 1 mL of 7% Na_2_Co_3_ solution was added to the mixture and incubated for 90 min. The absorbance was measured at 750 nm, and total phenolic content of BE was indicated as mg of gallic acid equivalents (GAE)/g dry weight. To measure total flavonoid in BE, 25 μL of BE was mixed to 160 μL of deionized water for 5 min. Subsequently, 7.5 μL of 5% NaNO_2_, 7.5 μL of 10% AlCl_3_, and 100 μL of 1M NaOH were added to the mixture and gently mixed. The absorbance was measured at 510 nm, and total flavonoid content was indicated as mg quercetin equivalents (QE)/g dry weight. All reagents for analysis were purchased from Sigma-Aldrich (St. Louis, MO, USA).

### 2.2. Cell Culture and the Sample Treatment

Caco-2 cells, the human colorectal adenocarcinoma cells obtained from ATCC (Manassas, VA, USA), were incubated in a humidified chamber at 37 °C with 5% CO_2_. The cells were maintained in Dulbecco’s modified Eagle’s medium supplemented with 10% fetal bovine serum, 100 U/mL of penicillin, 100 μg/mL of streptomycin, 1× vitamins, and 1× nonessential amino acids. Sterile water was used as the vehicle to dissolve the BE for the cell culture experiment. The Caco-2 cells were treated with 50 or 100 μg/mL of BE for 24 h. The cells were plated at a density of 0.5 × 10^6^ cells per well in a 12-well plate for qRT-PCR and 1.0 × 10^6^ cells per well in a 6-well plate for Western blot. The concentration of BE was determined by cell cytotoxicity using Cell Counting Kit-8 (Dojindo Molecular Technologies, Rockville, MD, USA), as previously described [44]. Briefly, the cells were treated with increasing concentrations (0–200 μg/mL) of BE for 24 h, and cell viability to the control without BE treatment was indicated as the cytotoxicity of BE. As the positive control, sodium dodecyl sulfate (0.5 mmol/L) exhibited near-zero viability, confirming the validation of the cytotoxicity assay. Cells incubated without BE served as a control for all experiments. All cell culture reagents were purchased from Hyclone (South Logan, UT, USA) unless indicated otherwise.

### 2.3. Quantitative Real-Time PCR (qRT-PCR)

The qRT-PCR analysis was conducted to analyze the intestinal gene expression in Caco-2 cells treated with BE. In brief, the concentrations of total RNA extracted by Trizol (Life Technologies, Carlsbad, CA, USA) were measured using Cytation 1 (BioTek, Winooski, VT, UST). The complementary strand of DNA (cDNA) was synthesized from RNA samples by reverse transcription. RNA was treated with DNase I (Invitrogen, Carlsbad, CA, USA) to remove contaminating genomic DNA and then reverse-transcribed using MMLV reverse transcriptase (Promega, Madison, WI, USA). SYBR green, a fluorescent dye that binds to double-stranded DNA, was used to quantify the reaction’s final products. The cycling conditions were 30 s at 95 °C, 40 cycles of 2 s at 95 °C, and 5 s at 60 °C, with a melt curve 65–95 °C with an increment of 0.5 °C for 5 s. CFX96 Touch Real-Time PCR detection system (Bio-Rad, Hercules, CA, USA) was used to analyze the qRT-PCR. The mRNA expression was calculated by the threshold cycle (Ct) value, the number of PCR cycles of fluorescent signal to reach the fixed threshold. The relative expression of the target gene to control was measured by using the 2^−ΔΔCt^ method. The primer sequences were designed by Beacon Designer (Premier Biosoft, Palo Alto, CA, USA), according to GenBank (Table 1). 

### 2.4. Western Blot Analysis

Western blot analyses were conducted to determine the protein levels in BE-treated Caco-2 cells as previously described [44]. The cell was lysed with Protease Inhibitor Cocktail (Merck, Kenilworth, NJ, USA) in cell lysis buffer (25 mM Tris-HCl pH 7.4, 150 mM NaCl, 1% Triton X-100). The concentration of cellular protein was measured using the Pierce BCA protein assay kit (ThermoFisher Scientific, Waltham, MA, USA). Fifty micrograms of cell protein was separated using 4–16% SDS-PAGE gel and subsequently transferred to a PVDF membrane. The membrane was blocked by 5% non-fat dry milk in Tris-buffered saline (TBS) solution containing the detergent Tween for 1 h, followed by incubation of primary antibody in TBS-Tween overnight at 4 °C. The blots were incubated with the appropriate secondary antibodies for 1 h and subsequently developed using a Supersignal west pico plus chemiluminescent substrate (ThermoFisher Scientific, Waltham, MA, USA) to detect horseradish peroxidase. Chemidoc XRS+ (Bio-Rad, Hercules, CA, USA) and Image Lab software (Bio-Rad, Hercules, CA, USA) were used for the analysis. The antibodies for scavenger receptor class B type 1 (SR-B1), Niemann-Pick C1-Like 1 (NPC1L1), ATP-binding cassette transporter A1 (ABCA1), sterol regulatory element-binding protein 2 (SREBP2), 3-hydroxy-3-methylglutaryl coenzyme A reductase (HMGR), LDL receptor (LDLR), proprotein convertase subtilisin/kexin type 9 (PCSK9), and ABCG8 were purchased from Abcam (Cambridge, MA, USA). β-actin obtained from Sigma (St. Louis, MO, USA) was used as a loading control to normalize the target protein. The antibodies used for Western blot are listed in Table 2.

### 2.5. LDL Uptake and Immunochemistry of LDLR

The uptake of LDL and the distribution of LDLR were assessed using the LDLR uptake assay kit (Abcam, Cambridge, MA, USA) following the manufacturer’s protocol. Briefly, Caco-2 cells were treated with BE for 24 h and were incubated with DyLight 550 fluorescent for an additional 4 h. The degree of LDL uptake was measured by excitation and emission at 540 nm and 570 nm, respectively. Next, the cells were fixed for 10 min and then incubated with a blocking solution for 30 min. Subsequently, the anti-LDLR antibody was incubated for 1 h, followed by Dylight 488-Conjugated secondary antibody for 1 h. The immunofluorescent staining of LDLR was analyzed using an AxioCam MRc camera (Carl Zeiss Microscopy, Jena, Germany).

### 2.6. Total Cholesterol Measurement

Total cholesterol levels in cells were measured as previously described [42,43]. Briefly, the cellular lipids were extracted with isopropanol. The cell debris from the isopropanol lipid extract was removed by centrifugation at 12,000× *g* for 5 min. Then, 100 μL of 10% Triton-X in chloroform was added to the extract and dried under N_2_. The lipids were solubilized in 240 μL of water. Cellular total cholesterol concentrations were measured enzymatically using a commercially available cholesterol kit (Asan Pharmaceutical Co., Seoul, Korea). After lipid extraction, the dried cell residues were hydrolyzed in 0.1 *n* NaOH, and protein levels were determined by BCA assay.

### 2.7. Statistical Analysis

One-way analysis of variance (ANOVA) and Newman–Keuls post hoc analysis were performed to detect significance between groups using GraphPad Prism 7 (GraphPad Software, La Jolla, CA, USA). *p* values less than 0.05 were indicated as statistically significant. All data are presented as mean ± S.E.M.

## 3. Results

### 3.1. Contents of Polyphenols in BE

The contents of total anthocyanin, phenolics, and flavonoids of BE were measured to examine the composition of polyphenols in BE. Total anthocyanin, phenolics, and flavonoids in BE, the standardized bilberry extract to 25% anthocyanins, exerted 237.9 ± 17.1 mg CGE/g, 338.5 ± 28.0 mg GAE/g, and 735.4 ± 18.2 mg QE/g, respectively (Table 3).

### 3.2. Cytotoxicity of BE in Caco-2 Cells

**Figure 1:**
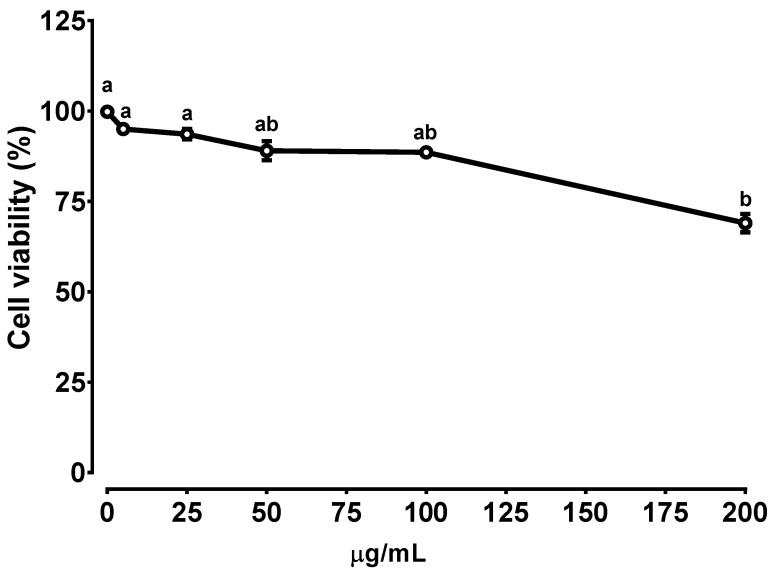
The cytotoxicity measurement of BE-treated Caco-2 cells. The cells were treated with increasing concentration (0–200 μg/mL) of BE for 24 h. Data points with different letters are significantly different (*p* < 0.05). Data are shown as means ± S.E.M. *n* = 6.

The cell viability of increasing concentrations of BE-treated Caco-2 cells was measured to investigate the cytotoxicity of BE. There was no significant difference in the viability of Caco-2 cells treated up to 100 μg/mL of BE (Figure 1). Therefore, the following experiments were conducted with a concentration of 50 or 100 μg/mL of BE.

### 3.3. Effects of BE on the Genes for Cholesterol Absorption

**Figure 2:**
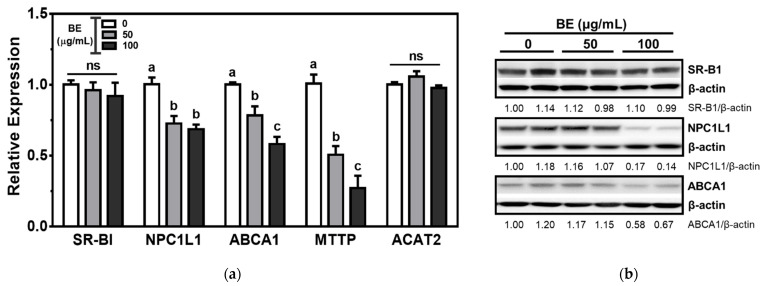
The genes involved in the intestinal cholesterol absorption in BE-treated Caco-2 cells. Cells were treated with 50 or 100 μg/mL of BE for 24 h. (**a**) The mRNA expression was measured by qRT-PCR analysis. Data are expressed as relative expressions to control. Bars with different letters are significantly different (*p* < 0.05), and ns indicates non-significance (*p* > 0.05). Values are means ± S.E.M. *n* = 6. (**b**) The Western blot was conducted twice, and the representative image is shown. β-actin was used as a loading control. Densitometry analysis was performed to measure the relative intensity, and the values are shown under each band.

The genes involved in intestinal cholesterol absorption were measured to investigate the effects of BE on cholesterol metabolism. The expression of SR-B1 was not altered by BE treatment. Compared to the untreated control cells, the mRNA abundance of NPC1L1, the apical transporter of cholesterol, was significantly (*p* < 0.05) decreased at both 50 and 100 μg/mL of BE-treated Caco-2 cells. Furthermore, the expression of ABCA1, the basolateral transporter of cholesterol, was significantly (*p* < 0.05) and concentration-dependently reduced by BE treatment. Chylomicron assembly is associated with intestinal cholesterol absorption. The expressions of genes involved in chylomicron assembly, i.e., microsomal triglyceride transfer protein (MTTP) and acetyl-CoA acetyltransferase 2 (ACAT2), were investigated. The expression of MTTP was significantly (*p* < 0.05) and dose-dependently decreased, whereas ACAT2 was not altered by BE treatment (Figure 2a). Consistent with mRNA expression, the protein levels of NPC1L1 and ABCA1 decreased in BE-treated Caco-2 cells. There was no alteration of SR-B1 protein levels by BEE treatment (Figure 2b).

### 3.4. Effects of BE on Cholesterol Biosynthesis and TICE

**Figure 3:**
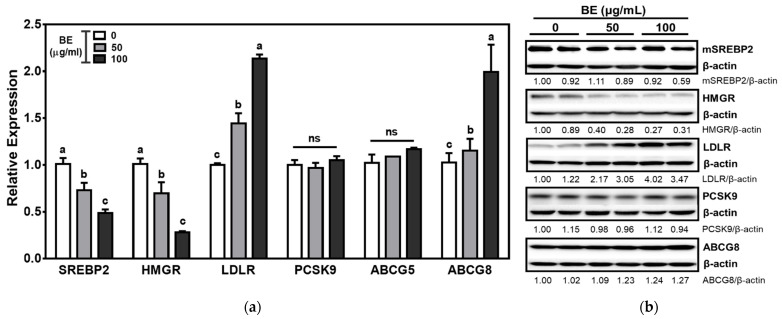
The genes involved in the intestinal cholesterol biosynthesis and TICE pathway in BE-treated Caco-2 cells. Cells were treated with 50 or 100 μg/mL of BE for 24 h. (**a**) The mRNA expression was measured by qRT-PCR analysis. Data are expressed as relative expressions to control. Bars with different letters are significantly different (*p* < 0.05), and ns indicates non-significance (*p* > 0.05). Values are means ± S.E.M. *n* = 6. (**b**) The Western blot was conducted twice, and the representative image is shown. β-actin was used as a loading control. Densitometry analysis was performed to measure the relative intensity, and the values are shown under each band.

Next, we measured whether BE can alter the genes related to cholesterol biosynthesis and the TICE pathway. SREBP2, the primary transcription factor for cholesterol biosynthesis and uptake, regulates HMGR and LDLR. The mRNA abundance of SREBP2 and HMGR was significantly (*p* < 0.05) and dose-dependently decreased by BE treatment. In contrast, the expression of LDLR, the receptor for uptake of LDL-derived cholesterol, was 2-fold increased in BE-treated Caco-2 cells. There was no alteration of the expression of PCSK9, the protease responsible for lysosomal degradation of LDLR, in BE-treated cells. ABCG5 and ABCG8 are the heterodimeric transporters present at the apical membrane of the enterocytes and accountable for the efflux of cholesterol back to the intestinal lumen for excretion. There was a significant (*p* < 0.05) and dose-dependent induction of ABCG8 expression by BE treatment (Figure 3a). Consistent with mRNA expression, the protein levels of SREBP2 and HMGR were attenuated in BE-treated cells. Furthermore, the protein levels of transporters for TICE, i.e., LDLR and ABCG8, were markedly increased by BE treatment. There were no changes in the protein levels of PCSK9 by BE treatment (Figure 3b).

### 3.5. Effects of BE on Cellular LDL Uptake, LDLR Distribution, and Cholesterol Levels

**Figure 4:**
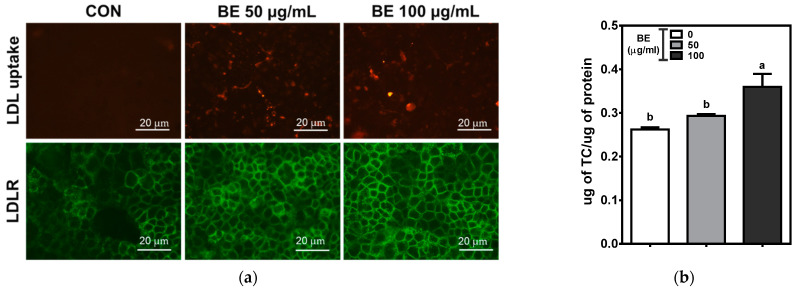
Cellular LDL uptake, LDLR distribution, and cholesterol levels in BE-treated Caco-2 cells. The cells were treated with 50 or 100 μg/mL of BE for 24 h. (**a**) LDL uptake was measured by additional incubation with Dylight 550 fluorescent label LDL for 4 h. Immunofluorescent staining of LDLR was measured using LDLR Dylight 488-conjugated secondary antibody. Scale bar: 20 μm. (**b**) Cellular total cholesterol concentration was measured enzymatically in the lipid extract of the cells. Bars with different letters are significantly different (*p* < 0.05). Values are means ± S.E.M. *n* = 6.

The mRNA and protein levels of LDLR were markedly increased in BE-treated cells. We further examined the effects of BE on cellular LDL uptake using Dylight 550 conjugated LDL in Caco-2 cells. The increased uptake of LDL was observed by 100 μg/mL of BE treatment. Consistent with Western blot results, LDLR distribution measured by immunostaining was noticeably increased by 50 and 100 μg/mL of BE treatment compared to control (Figure 4a). Furthermore, total cholesterol levels were significantly (*p* < 0.05) increased in 100 μg/mL of BE treatment (Figure 4b).

### 3.6. Effects of BE on Fatty Acid Metabolism

**Figure 5:**
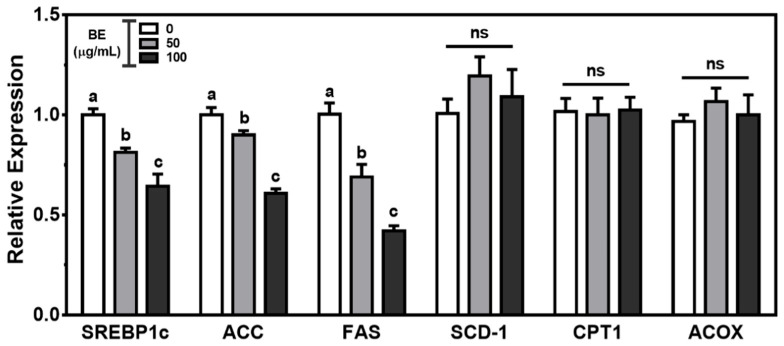
The genes involved in the fatty acid metabolism in BE-treated Caco-2 cells. The cells were treated with 50 or 100 μg/mL of BE for 24 h. The mRNA expression was measured by qRT-PCR analysis. Data are expressed as relative expressions to control. Bars with different letters are significantly different (*p* < 0.05), and ns indicates non-significance (*p* > 0.05). Values are means ± S.E.M. *n* = 6.

Next, we measured the expression of genes for lipogenesis and fatty acid oxidation to investigate the effect of BE on fatty acid metabolism. BE significantly (*p* < 0.05) and dose-dependently decreased the mRNA level of SREBP1c, the lipogenic transcription factor, with a concomitant decrease in the downstream genes, i.e., acetyl-CoA carboxylase (ACC) and fatty acid synthase (FAS). However, there was no significant alteration in the expression of carnitine palmitoyltransferase 1 (CPT1) and acyl-CoA oxidase (ACOX), the enzymes for mitochondrial and peroxisomal fatty acid oxidation, respectively, by BE treatment (Figure 5).

### 3.7. Effects of BE on the Regulation of Sirtuins

**Figure 6:**
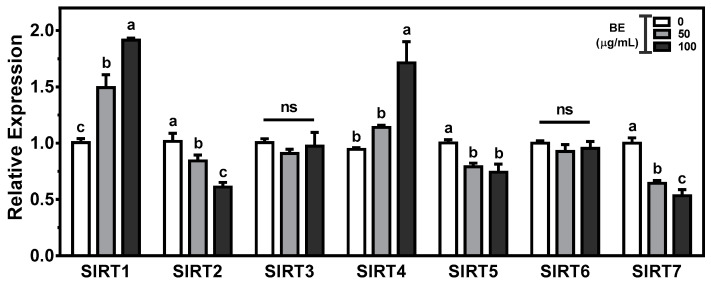
The expression of sirtuins (SIRTs) in BE-treated Caco-2 cells. The cells were treated with 50 or 100 μg/mL of BE for 24 h. The mRNA expression was measured by qRT-PCR analysis. Data are expressed as relative expressions to control. Bars with different letters are significantly different (*p* < 0.05), and ns indicates non-significance (*p* > 0.05). Values are means ± S.E.M. *n* = 6.

Sirtuins(SIRTs) play key aspects in the regulation of metabolic homeostasis, including cholesterol metabolism. To gain insight into the effects of BE on intestinal cholesterol metabolism, we measured the expression of seven sirtuin isoforms in BE-treated Caco-2 cells. There was a significant (*p* < 0.05) and dose-dependent induction of SIRT1 expression in BE-treated Caco-2 cells. The mRNA abundance of SIRT4 was significantly (*p* < 0.05) augmented in 100 μg/mL of BE-treated cells. In contrast, significant (*p* < 0.05) and dose-dependent decreases in the expression of SIRT2 and SIRT7 were observed in BE-treated Caco-2 cells. The significantly (*p* < 0.05) reduced abundance of SIRT5 was observed by 100 μg/mL of BE. However, the expressions of SIRT3 and SIRT6 were not altered by BE treatment (Figure 6).

## 4. Discussion

Regulation of cholesterol homeostasis is one of the modifiable factors to reduce CVD prevalence [48]. The liver and the intestine play prominent roles in the regulation of cholesterol net balance in the plasma [49]. Reverse cholesterol transport mediated cholesterol excretion is the well-accepted route for cholesterol elimination from the body [50,51]. Most studies about the effects of natural products on hypercholesterolemia are focused on the liver, the primary site for cholesterol metabolism. However, emerging research suggests that intestinal enterocytes play an important part in the direct disposal of plasma-derived cholesterol [52]. Studies revealed the existence of the nonbiliary TICE pathway in mice, rats, dogs, and humans [11,12,13,53,54,55,56,57]. The fecal cholesterol elimination is effectively regulated by the dynamic interaction of the hepatobiliary cholesterol excretion pathway and nonbiliary TICE pathway [58]. However, the relative contribution of TICE to cholesterol excretion was different under normal, pharmacological, and pathophysiological conditions. Hepatobiliary cholesterol excretion requires biliary secretion that is associated with gallstone formation [59]. In addition, the molecular mechanisms underlying this classical cholesterol excretion pathway are well known, whereas the mechanism underlying the TICE pathway remains largely elusive. Therefore, stimulation of TICE provides a novel and promising target to increase cholesterol elimination without side effects. Several studies reported that polyphenol-rich natural products such as black chokeberry, blackcurrant, black elderberry, and resveratrol, as well as casein-derived peptides, induced the TICE pathway [42,43,44,45,60].

Bilberry and anthocyanin supplementation has been shown to ameliorate hyperlipidemia in both animals and humans [39,40,41,61]. To elucidate the mechanisms by which BE regulates intestinal cholesterol metabolism, we evaluated whether BE could modulate the genes involved in cholesterol flux, chylomicron formation, cholesterol biosynthesis, and TICE. Cholesterol absorption is mediated by intestinal cholesterol transporters, i.e., NPC1L1, SR-B1, and ABCA1 [62,63,64,65]. The mRNA and protein levels of NPClL1 were significantly decreased in BE-treated Caco-2 cells. NPC1L1, the apical transporter of enterocytes, is responsible for the absorption of cholesterol in the intestine [63,66]. Ezetimibe, the potent cholesterol absorption inhibitor that targets NPC1L1, is one of the effective medications for hypocholesterolemia [67]. Several studies reported that the effect of ezetimibe on cholesterol excretion might be associated with increasing TICE for cholesterol removal [17]. Consistent with our findings, polyphenols and polyphenol-rich extracts, including luteolin, curcumin, wild *Lonicera caerulea* berry extract, black chokeberry extract, blackcurrant extract, and black elderberry extract, inhibited NPClL1 while simultaneously decreasing cholesterol uptake [68]. Therefore, the reduction in NPC1L1 in BE-treated cells demonstrates the potential of BE in the prevention of hypercholesterolemia. ABCA1, the transporter protein present in the basolateral membrane of the enterocyte, facilitates the efflux of cholesterol to the intestinal lumen [64,65,69]. There were significant decreases in both mRNA and protein of ABCA1 in Caco-2 cells treated with BE. Several studies reported the reduction in intestinal ABCA1 by polyphenol-abundant foods had hypocholesterolemic effects [42,43]. Consistent with the present study, polyphenols reduced cholesterol absorption by interacting with cholesterol transporters and carriers located at the brush border membrane [21]. The availability of cholesterol, the substrate for chylomicron, has an effect on both assembly and secretion of intestinal chylomicron. Chylomicron formation and transport are highly associated with cholesterol absorption [70]. ACAT2, the cholesterol esterification enzyme, provides cholesterol esters necessary for chylomicron assembly, consequently increasing the efficiency of cholesterol absorption [71]. MTTP plays a central role in lipoprotein metabolism, and its inhibitors decrease plasma LDL cholesterol levels by preventing the assembly of apoB-containing lipoprotein [72]. We observed a significant and dose-dependent reduction in MTTP in BE-treated cells. These results indicate that BE may exert an inhibitory effect on cholesterol absorption by altering the expression of genes involved in cholesterol transporters and chylomicron assembly.

De novo cholesterol synthesis is one of the regulatory feedback mechanisms that control the maintenance of cholesterol homeostasis [2,3]. SREBP2, the transcription factor for cholesterol metabolism, regulates cholesterol homeostasis by activating HMGR and LDLR [73,74]. HMGR, the enzyme involved in the committed step of cholesterol synthesis, has been targeted to lower plasma cholesterol levels [75,76]. Statin, the inhibitor of HMGR, is the most commonly prescribed medication for the prevention of CVD [77]. The mRNA and protein levels of SREBP2 and HMGR were reduced by BE treatment. Several polyphenols are reported to have the HMG-CoA binding site and act as competitive inhibitors of substrate binding on HMGR [78]. These results support that the hypocholesterolemic effects of polyphenols and polyphenol-abundant products may be attributed to their potential role as an inhibitor of HMGR.

To elucidate the mechanisms behind TICE, several groups have been established to identify the source of cholesterol and the intestinal receptors required for the TICE flux. Intestinal SR-B1-mediated uptake of HDL-derived cholesterol is not likely involved in fecal neutral sterol excretion through the TICE pathway [79]. In contrast, several studies reported that stimulation of LDR increased the TICE and suggested that other receptors in the LDLR family involved in the uptake of apoB-containing lipoprotein-derived cholesterol may increase TICE [52]. Therefore, natural products or medications that can stimulate the intestinal receptor responsible for increasing the flux of apoB-lipoprotein derived cholesterol have the potential to open new preventative targets for hypercholesterolemia. In the present study, BE markedly upregulated both mRNA and proteins levels of intestinal LDLR with a concomitant increase in LDL cholesterol uptake. LDLR, the receptor responsible for plasma LDL uptake, is the downstream target gene of SREBP2 that responds to intracellular cholesterol levels. Significant induction of LDLR in BE-treated Caco-2 cells was independent of the transcriptional regulation of SREBP2. PCSK9, the posttranslational regulator of LDLR by mediating lysosomal degradation, has been reported to repress the TICE pathway [80,81,82]. To address the molecular mechanisms of marked induction of LDLR by BE, we measured the mRNA and protein levels of PCSK9 in BE-treated Caco-2 cells. There was no alteration of PCSK9 in BE-treated Caco-2 cells. These results indicate that PCSK9 was not responsible for the alteration of LDLR in response to BE, and the significant upregulation of LDLR expression by BE treatment is likely to be mediated by an unknown transcription factor. ABCG5/G8, the functional heterodimers present at the apical membrane of the enterocyte, are responsible for the secretion of cholesterol and sterols [64]. Studies reported that intestinal ABCG5/G8 significantly contributes to TICE, and the expression of ABCG5/G8 altered by LXR also increased TICE [13,14]. There were increases in mRNA and protein levels of ABCG8 in BE-treated cells. Consistent with our results, soluble dietary fiber upregulated the mRNA level of ABCG8 but not that of ABCG5 in Caco-2 cells [83]. Polyphenols and polyphenol-abundant natural products increased the ABCG8 levels in Caco-2 cells [42,44,84]. These results indicate LDLR-mediated cholesterol uptake can be translocated to the intestinal lumen via ABCG5/G8 involved stimulation of TICE.

The formation of lipoprotein is dependent on the availability of substrate for triglyceride synthesis [70]. To address the effects of BE on fatty acid metabolism, we investigated the genes involved in lipogenesis and fatty acid β-oxidation in BE-treated Caco-2 cells. The expression of the genes for lipogenesis, i.e., SREBP1c, ACC, and FAS, was attenuated by BE treatment in a dose-dependent manner. However, the genes for fatty acid β-oxidation were not altered by BE treatment. SREBP1c is the critical transcription factor for de novo lipogenesis and activates major biosynthetic enzymes for fatty acids, i.e., ACC, FAS, and stearoyl-CoA desaturase-1 (SCD-1) [85]. Similar to our results, polyphenols in red wine inhibited the secretion of the specific marker of intestinal chylomicron particles, apolipoprotein B48, in Caco-2 cells [86,87]. These results suggest that lipogenic gene alteration by BE may ameliorate chylomicron secretion in the intestine. Further study is warranted to investigate the effect of BE on lipogenesis and chylomicron assembly.

All of the gene changes indicate that BE altered the genes for cholesterol and lipid metabolism. The seven mammalian SIRTs are NAD+ dependent deacetylases that play significant roles in cellular metabolism and epigenetic modification [88]. SIRTs have been shown to act as regulators in cholesterol and lipid metabolism via different and distinct mechanisms [89]. SIRT1 is a positive regulator of LXR, which is involved in the regulation of cholesterol homeostasis [90]. Resveratrol, a polyphenol found in abundant amounts in grapes and red wines, is a natural SIRT1 activator [91]. However, little is known about the effects of polyphenols on the alteration of SIRTs. There were significant increases in SIRT1 and SIRT3 and decreases in SIRT2, SIRT5, and SIRT7 expression in BE-treated cells. Further study is required to elucidate the mechanisms by which BE alters SIRTs.

## 5. Conclusions

In conclusion, BE modulated the genes involved in intestinal cholesterol metabolism, i.e., cholesterol absorption and biosynthesis, chylomicron formation, and TICE, in Caco-2 cells. Further research is needed to address the underlying mechanisms responsible for the effect of BE on stimulation of TICE in vitro and in vivo, which can translate the changes of genes by BE observed in the present study to the physiological significance. In addition, another study is underway in which bioactive components in BE are responsible for stimulating TICE and altering SIRTs. The present study supports the potential of cholesterol-lowering effects of BE in the prevention of CVD, which may be attributed to the alteration of intestine-specific stimulation of TICE.

## Figures and Tables

**Table 1:** The primer sequences for qRT-PCR analysis.

Genes	GenBank No.	Forward (5′ to 3′)	Reverse (5′ to 3′)
SR-B1	NM_005505.5	AGAATAAGCCCATGACCCTGAA	CGCCGAGGGTGGTGAA
NPC1L1	NM_013389.3	CACTGGATCACTCGAGGTGTTG	CCAGTCCCACGCTGATGTG
ABCA1	NM_005502	TTTCTCAGACAACACTTGACCAAGTA	GGTTTTTGTGTAATGAGAGGTCTTTTAA
MTTP	NM_000253.4	TCCCCGTTCGGCATCTAC	CTTAGAATGCCAGAACCCGAGTA
ACAT2	NM_005891	TGGGCCACCCTCTTGGA	CCAGTGTGTGTAACAGGGTCACA
SREBP2	BC056158.1	TCCGCCTGTTCCGATGTAC	TGCACATTCAGCCAGGTTCA
HMGR	NM_000859.3	CCCAGTTGTGCGTCTTCCA	TTCGAGCCAGGCTTTCACTT
LDLR	AY114155.1	ACTGGGTTGACTCCAAACTTCAC	GGTTGCCCCCGTTGACA
PCSK9	NM_174936.3	TTCCTGGTGAAGATGAGT	TTCCTGGTGAAGATGAGT
ABCG5	NM_022436.3	GCGTAGGTCTCCTTTACCAGTTTG	GGAAACAGATTCACAGCGTTCA
ABCG8	NM_022437.3	GCCGCCCTCTTGTTCATG	TAACATTTGGAGATGACATCCAGAA
SREBP1c	NM-001005291	TCAGCGAGGCGGCTTTGGAGCAG	CATGTCTTCGATGTCGGTCAG
ACC	BC137287.1	GGATCCGGCGCCTTACTT	CTCCGATCCACCTCATAGTTGAC
FAS	AY451392.1	CGCTCGGCATGGCTATCT	CTCGTTGAAGAACGCATCCA
SCD-1	NM_005063	CCGACGTGGCTTTTTCTTCT	TGGGTGTTTGCGCACAAG
CPT1	NM_001876.4	TTATCGCCAAGGATGGCTCTA	CCACACCATCACCCCAAGA
ACOX	BC008767.2	CTTGCTTCACCAGGCAACTG	TTCCAGGCGGGCATGA
SIRT1	NM_012238.4	TAGTTCTTGTGGCAGTAA	CATCAGGCTCATCTTCTA
SIRT2	NM_012237.3	AACCATCTGTCACTACTT	TATCTATGTTCTGCGTGTA
SIRT3	NM_012239.5	GCTCCCAGTTTCTTCTTT	CCACTTCCAACAACACTT
SIRT4	NM_012240.2	CTTCATCACCCTTTCCAA	ACCTGTAGTCTGGTATCC
SIRT5	NM_012241.3	AAGCACATAGTCATCATCT	TTCTCCAATAACCTCCAG
SIRT6	NM_016539.2	AGGGACAAACTGGCAGAG	TGTGTCTCGGACGTACTG
SIRT7	NM_016538.2	AATACTTGGTCGTCTACAC	TGTCCACACTCCATTAGG
GAPDH	NM_002046.7	GGTGGTCTCCTCTGACTTCAACA	GTTGCTGTAGCCAAATTCGTTGT

The product size of each gene is 100 bp.

**Table 2:** Antibodies used for Western blot.

Antibodies	Company	Catalog No.
Rabbit polyclonal Anti-Scavenging Receptor SR-BI antibody	Abcam	ab106572
Rabbit monoclonal Anti-Niemann Pick C1-Like 1 antibody	Abcam	ab124801
Mouse monoclonal Anti-ABCA1 antibody	Abcam	ab18180
Rabbit polyclonal Anti-SREBP2 antibody	Abcam	ab30682
Rabbit monoclonal Anti-HMGCR antibody	Abcam	ab174830
Rabbit monoclonal Anti-LDL Receptor antibody	Abcam	ab52818
Goat polyclonal Anti-PCSK9 antibody	Abcam	ab28770
Rabbit polyclonal Anti-ABCG8 antibody	Abcam	ab126493
Monoclonal Anti-β-Actin antibody	Sigma	A5441
Goat anti-Mouse IgG (H + L) secondary antibody, HRP	Invitrogen	31430
Goat anti-Rabbit IgG (H + L) secondary antibody, HRP	Invitrogen	31460
Rabbit anti-Goat IgG (H + L) secondary antibody, HRP	Invitrogen	31402

**Table 3:** Polyphenol contents in BE.

	Total Anthocyanin (mg CGE/g)	Total Phenolics (mg GAE/g)	Total Flavonoid (mg QE/g)
Anthocyanin-rich bilberry extract (BE)	237.9 ± 17.1	338.5 ± 28.0	735.4 ± 18.2

Data represent mean ± SEM. CGE, cyanidin-3-glucoside equivalents; GAE, gallic acid equivalents; QE, quercetin equivalents.

## Data Availability

The data presented in this study are available in the article.
